# Running Gait and Control of Quadruped Robot Based on SLIP Model

**DOI:** 10.3390/biomimetics9010024

**Published:** 2024-01-03

**Authors:** Xiaolong He, Xinjie Li, Xiangji Wang, Fantuo Meng, Xikang Guan, Zhenyu Jiang, Lipeng Yuan, Kaixian Ba, Guoliang Ma, Bin Yu

**Affiliations:** 1 School of Mechanical Engineering, Yanshan University, Qinhuangdao 066004, China; hxl@ysu.edu.cn (X.H.); lxjlxj@stumail.ysu.edu.cn (X.L.); 21b908048@stu.hit.edu.cn (X.W.); mengft@stumail.ysu.edu.cn (F.M.); gxk@stumail.ysu.edu.cn (X.G.); for0207@126.com (Z.J.); bkx@ysu.edu.cn (K.B.); magl@ysu.edu.cn (G.M.); 2 School of Mechatronics Engineering, Harbin Institute of Technology, Harbin 150001, China; hitylp@126.com

**Keywords:** legged robot, high-speed running, SLIP model, running gait

## Abstract

Legged robots have shown great adaptability to various environments. However, conventional walking gaits are insufficient to meet the motion requirements of robots. Therefore, achieving high-speed running for legged robots has become a significant research topic. In this paper, based on the Spring-Loaded Inverted Pendulum (SLIP) model and the optimized Double leg—Spring-Loaded Inverted Pendulum (D-SLIP) model, the running control strategies for the double flying phase Bound gait and the Rotatory gallop gait of quadruped robots are designed. First, the dynamics of the double flying phase Bound gait and Rotatory gallop gait are analyzed. Then, based on the “three-way” control idea of the SLIP model, the running control strategy for the double flying phase Bound gait is designed. Subsequently, the SLIP model is optimized to derive the D-SLIP model with two touchdown legs, and its dynamic characteristics are analyzed. And the D-SLIP model is applied to the running control strategy of the Rotatory gallop gait. Furthermore, joint simulation verification is conducted using Adams virtual prototyping and MATLAB/Simulink control systems for the designed control strategies. Finally, experimental verification is performed for the double flying phase Bound gait running control strategy. The experimental results demonstrate that the quadruped robot can achieve high-speed and stable running.

## 1. Introduction

Compared to wheeled robots [1,2] and tracked robots [3,4], legged robots have exhibited excellent adaptability in unknown and unstructured environments [5]. They are particularly suited for tasks such as exploration, transportation, rescue operations, and military assistance in complex outdoor environments, making them a focal point of research for roboticists worldwide [6,7,8,9]. However, the movement speed of existing legged robots falls far short of that of natural quadruped mammals. For instance, cheetahs can reach speeds of up to 110 km/h [10]. Thus, the capability of high-speed running for legged robots has become a critical and challenging problem in the research area of legged robots. Achieving high-speed running in legged robots requires breakthroughs in both appropriate mechanical structures and stable control algorithms. Scholars worldwide have conducted extensive research to meet the demands of high-speed running for legged robots. And a series of legged robot prototypes have been launched, such as the WildCat robot [11] developed by Boston Dynamics, the MIT (Massachusetts Institute of Technology) Cheetah 3 robot [12] and Mini Cheetah robot [13,14] developed by the MIT [15], and the Parallel Actuated Pantograph Leg for High-speed robot developed by our team [16].

During the process of walking or running, quadruped mammals swing their legs in a certain pattern, creating intermittent support between the foot and the ground. This leg movement pattern is referred to as the gait. Common dynamic gaits for quadruped mammals include Walk gait, Trot gait, Bound gait, and Gallop gait [17]. Based on speed, gaits can be categorized as medium- to high-speed gaits, represented by the Bound gait and Gallop gait, and medium- to low-speed gaits, represented by the Trot gait. Consequently, this paper focuses on the analysis of the medium- to high-speed gaits, including the double flying phase Bound gait and the Rotary gallop gait for quadruped robots, and then designs their control strategy.

The SLIP model is a simplified model effectively describing the movement characteristics of quadruped mammals. This model serves as a mechanism model for single-leg control of running robots and compared to the LIP (Linear Inverted Pendulum) model, is more closely related to the elastic muscle-rich mammalian leg. It can explain the buffering mechanism for impact forces at the foot–ground contact [18]. Building on the SLIP model, Raibert developed the well-known heuristic three-part controller by fully utilizing its self-stabilization properties [19,20]. There is a wealth of literature on modeling, running gaits design, and control of legged robots based on the SLIP model. Guo [21] proposed a control strategy combining the virtual SLIP model and active forces. Thanh [16] introduced a biped robot mimicking SLIP with inverters to improve running performance. Tseng [22] proposed a rigid body and two eccentric spring-loaded inverted pendulum (eSLIP) legs with dampers and designed a model-based strategy for a quadruped robot with differentiated fore- and hind-leg ground reaction force patterns to generate animal-like running behavior. Yu [23] presented a two-layered Dual-SLIP model-based Task-space Formulation (DS-TSF) to control the 12-DoF(Degree of Freedom) quadruped robot with an active spine. Patrick M. [24] proposed a 3D spring-loaded inverted pendulum (SLIP) template model for high-speed motion control of humanoid robots. Wu [25] devised a novel closed-chain elastic-bionic leg (CEL) with one actuated degree of freedom (DoF) based on SLIP and demonstrated running motion on a treadmill in a laboratory setting. Karagoz [26] proposed a spring-mass model with a trunk based on the virtual pendulum concept. Han [27] proposed a 3D-HFC control strategy based on the classic Raibert controller.

While the traditional SLIP model, employing Raibert’s “virtual leg” concept, can be directly applied to the Trot gait of quadruped robots, it cannot be directly applied to high-speed running gaits. To achieve high-speed running in the Bound gait, it is necessary to delve deeper into the control strategy for velocity, body height, and body pitch angle based on the SLIP model’s control strategy approach for Bound gait. Additionally, due to the asymmetry of the Gallop gait, the traditional SLIP model and control strategies are not applicable. Therefore, it is necessary to optimize the existing model to design a mechanism model that can be directly applied to the Gallop gait. And, based on it, the Gallop gait motion control strategy is designed.

This paper investigates the dynamic characteristics and control strategies of two high-speed running gaits: the double flying phase Bound gait based on the SLIP model and the Rotary gallop gait based on the D-SLIP model. The structure of the paper is as follows: Section 2 analyzes the dynamic characteristics of the double flying phase Bound gait and the Rotary gallop gait; Section 3 designs the running control strategies of the double flying phase Bound gait and the Rotary gallop gait; Section 4 conducts joint simulation verification of the two high-speed running gaits using Adams virtual prototyping and the MATLAB/Simulink control system; Section 5 presents the experimental verification; Section 6 concludes the paper.

## 2. Dynamic Analysis of Quadruped Robot High-Speed Running Gaits

### 2.1. Leg Mechanical Structure and Actuators

**Figure 1:**
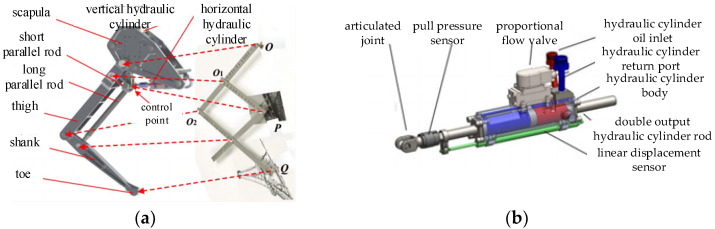
Robot leg structure and actuator. (**a**) Robot leg structure. (**b**) Highly integrated hydraulic drive unit.

Inspired by the concept of a traditional drafting tool called “pantograph”, a high-speed robot leg mechanical structure has been designed [16], as shown in Figure 1a. High-speed running robots exhibit rapid motion, high leg swinging frequencies, significant ground impacts, and the need for substantial leg output forces. It requires a driving element with a high driving force, fast response, and small volume. Consequently, the leg structure in this prototype employs a double-link symmetrical hydraulic driving unit as the actuator, as shown in Figure 1b.

### 2.2. Dynamic Analysis of the Double Flying Phase Bound Gait

**Figure 2:**
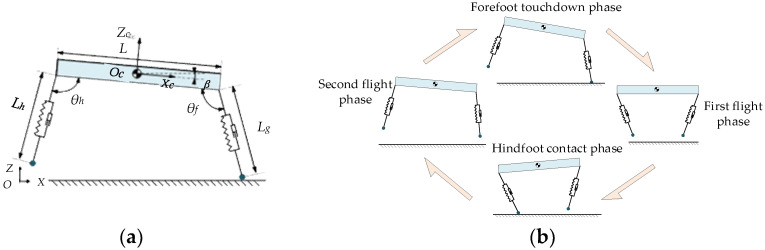
Double flying phase Bound gait motion analysis. (**a**) Robot planar three-bar model. (**b**) Double flying phase Bound gait schematic diagram.

The Bound gait is a symmetric gait, with two front legs and two hind legs forming a pair each, simultaneously touching and leaving the ground. The concept of virtual legs introduced by Raibert [28] can be employed to further simplify the dynamic model in the sagittal plane in the Bound gait, which is eventually simplified to a planar three-bar model in the sagittal plane, as shown in Figure 2a. The double flying phase Bound gait offers greater extension, higher speed, and more complex dynamic characteristics, making it suitable for achieving high-speed running in legged robots. Based on the dynamic characteristics of each motion state, the double flying phase Bound gait is divided into four phases: the first flying phase; the hind leg touch-down phase; the second flying phase; and the front leg touch-down phase. The dynamic characteristics of each phase are used as criteria to determine the different phases, as shown in Figure 2b.

The dynamic mathematical expressions for different phases of the double flying phase Bound gait are as follows.

State equation for the first flying phase are as follows:

(1) $$\{\begin{array}{c} \begin{array}{c} \ddot{x}=0\\ \ddot{z}=-g \end{array}\\ \ddot{\beta }=0 \end{array}$$

State equation for the front leg touch-down phase are as follows:

(2) $$\{\begin{array}{c} m\ddot{x}-\left(F_{kf}+F_{cf}+F_{thrustf}\right)\cos\left(\theta_{f}+\beta \right)+\tau_{f}\sin\left(\theta_{f}+\beta \right)/l_{f}=0\\ m\ddot{y}+\left(F_{kf}+F_{cf}+F_{thrustf}\right)\sin\left(\theta_{f}+\beta \right)+\tau_{f}\cos\left(\theta_{f}+\beta \right)/l_{f}-mg=0\\ J\ddot{\beta }-\left(F_{kf}+F_{cf}+F_{thrustf}\right)L\sin\theta_{f}/2-\left(1-L\cos\theta_{f}/2l_{f}\right)\tau_{f}=0 \end{array}$$

State equation for the second flying phase are as follows:

(3) $$\{\begin{array}{c} \begin{array}{c} \ddot{x}=0\\ \ddot{z}=-g \end{array}\\ \ddot{\beta }=0 \end{array}$$

State equation for the hind leg touch-down phase are as follows:

(4) $$\{\begin{array}{c} m\ddot{x}-\left(F_{kh}+F_{ch}+F_{thrusth}\right)\cos\left(\theta_{h}-\beta \right)+\tau_{h}\sin\left(\theta_{h}-\beta \right)/l_{h}=0\\ m\ddot{y}+\left(F_{kh}+F_{ch}+F_{thrusth}\right)\sin\left(\theta_{h}-\beta \right)+\tau_{h}\cos\left(\theta_{h}-\beta \right)/l_{h}-mg=0\\ J\ddot{\beta }+\left(F_{kh}+F_{ch}+F_{thrusth}\right)L\sin\theta_{h}/2-\left(1-Lcos\theta_{h}/2l_{h}\right)\tau_{h}=0 \end{array}$$

Among them:

(5) $$\left\{ \begin{array}{l} F_{kh}=k\left(L_{0}-l_{h}\right)\\ F_{kf}=k\left(L_{0}-l_{f}\right)\\ F_{ch}=k{\dot{l}}_{h}\\ F_{cf}=k{\dot{l}}_{f} \end{array}\right.$$

In the equations, $F_{thrusth}$ is the active force of the hind leg (N); $F_{kh}$ is the spring force of the hind leg (N); $F_{ch}$ is the damping force of the hind leg (N); $\tau_{h}$ is the active moment of the hind leg hip joint (N·m); $F_{thrustf}$ is the active force of the front leg (N); $F_{kf}$ is the spring force of the front leg (N); $F_{cf}$ is the damping force of the front leg (N); $\tau_{f}$ is the active moment of the front leg hip joint (N·m); $l_{h}$ is the actual length of the hind leg (m); $l_{f}$ is the actual length of the front leg (m); J is the moment of inertia of pitching motion; $L_{0}$ is the original length of the leg (m); $\theta_{f}$ is the angle between the front leg and the body (rad); $\theta_{h}$ is the angle between the hind leg and the body (rad); k is the stiffness of the leg (N/m); β is the pitch angle (rad); m is the weight of the body (kg); x is the horizontal displacement (m); y is the vertical displacement; z is the vertical displacement (m); L is the length of the body (m).

### 2.3. Dynamic Analysis of Rotatory Gallop Gait

**Figure 3:**
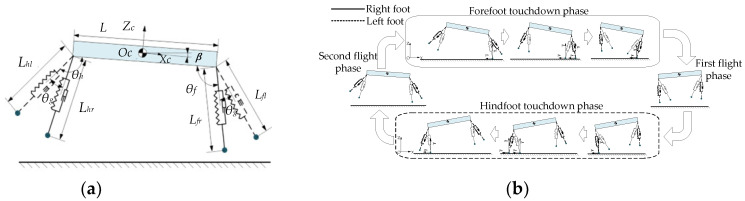
Rotatory gallop gait motion analysis. (**a**) Robot planar-bar model. (**b**) Schematic diagram of Rotatory gallop gait motion.

In contrast to the Bound gait, the Rotatory gallop gait is an asymmetric gait, with different movement states of the legs on the same side. Ignoring the roll and yaw angles of the fuselage can further simplify the dynamic model of the robot to a planar five-bar model, as shown in Figure 3a. The Rotatory gallop gait can be divided into four phases during the running process: the front leg touching the ground support stage (the front leg touch-down phase); the aerial stage in which the legs swing to the inside of the body after the front leg leaves the ground (the first flying phase); the hind leg touching the ground support stage (the hind leg touch-down phase); and the aerial stage in which the legs swing to the outside of the body after the hind leg leaves the ground (the second flying phase). The characteristics of each phase are used as criteria to determine the different phases for orderly transitions between the four motion phases of the Rotatory gallop gait. In the touch-down phase, the leg making initial contact with the ground is called the leading leg, while the leg making contact later is called the following leg. The motion phases of the Rotatory gallop gait are shown in Figure 3b.

The dynamic mathematical expressions for different phases of the Rotatory gallop gait are as follows:

The first flying phase are as follows:

(6) $$\{\begin{array}{c} \begin{array}{c} \ddot{x}=0\\ \ddot{z}=-g \end{array}\\ \ddot{\beta }=0 \end{array}$$

The front leg touch-down phase ar as follows:

(1) Leading leg touches down are as follows:

(7) $$\{\begin{array}{c} m\ddot{x}-\left(F_{kfr}+F_{cfr}+F_{thrustfr}\right)\cos\left(\theta_{f}+\beta \right)+\tau_{fr}\sin\left(\theta_{f}+\beta \right)/l_{fr}=0\\ m\ddot{y}+\left(F_{kfr}+F_{cfr}+F_{thrustfr}\right)\sin\left(\theta_{f}+\beta \right)+\tau_{fr}\cos\left(\theta_{f}+\beta \right)/l_{fr}-mg=0\\ J\ddot{\beta }-\left(F_{kfr}+F_{cfr}+F_{thrustfr}\right)L\sin\theta_{f}/2-\left(1-L\cos\theta_{f}/2l_{f}\right)\tau_{f}=0 \end{array}$$

(2) Both legs touch down are as follows:

(8) $$\{\begin{array}{c} \begin{array}{l} m\ddot{x}-\left(F_{kfr}+F_{cfr}+F_{thrustfr}\right)\cos\left(\theta_{f}+\beta \right)-\left(F_{kfl}+F_{cfl}+F_{thrustfl}\right)\cos\left(\theta_{f}+\theta_{g}+\beta \right)\\ +\tau_{fr}\sin\left(\theta_{f}+\beta \right)/l_{fr}+\tau_{fl}\sin\left(\theta_{f}+\theta_{g}+\beta \right)/l_{fl}=0 \end{array}\\ \begin{array}{l} m\ddot{y}+\left(F_{kfr}+F_{cfr}+F_{thrustfr}\right)\sin\left(\theta_{f}+\beta \right)+\left(F_{kfl}+F_{cfl}+F_{thrustfl}\right)\sin\left(\theta_{f}+\theta_{g}+\beta \right)\\ +\tau_{fr}\cos\left(\theta_{f}+\beta \right)/l_{fr}+\tau_{fl}\cos\left(\theta_{f}+\theta_{g}+\beta \right)/l_{fl}-mg=0 \end{array}\\ \begin{array}{l} J\ddot{\beta }-\left(F_{kfr}+F_{cfr}+F_{thrustfr}\right)L\sin\theta_{f}/2-\left(F_{kfl}+F_{cfl}+F_{thrustfl}\right)L\sin\left(\theta_{f}+\theta_{g}\right)/2\\ -\tau_{fr}\left(1-Lcos\theta_{f}/2l_{fr}\right)-\tau_{fl}\left[1-Lcos\left(\theta_{f}+\theta_{g}\right)/2l_{fl}\right]=0 \end{array} \end{array}$$

(3) Following leg touches down are as follows:

(9) $$\{\begin{array}{c} m\ddot{x}-\left(F_{kfl}+F_{cfl}+F_{thrustfl}\right)\cos\left(\theta_{f}+\theta_{g}+\beta \right)+\tau_{fl}\sin\left(\theta_{f}+\theta_{g}+\beta \right)/l_{fl}=0\\ m\ddot{y}+\left(F_{kfl}+F_{cfl}+F_{thrustfl}\right)\sin\left(\theta_{f}+\theta_{g}+\beta \right)+\tau_{fl}\cos\left(\theta_{f}+\theta_{g}+\beta \right)/l_{fl}-mg=0\\ J\ddot{\beta }-\left(F_{kfl}+F_{cfl}+F_{thrustfl}\right)L\sin\left(\theta_{f}+\theta_{g}\right)/2-\tau_{fl}\left[1-Lcos\left(\theta_{f}+\theta_{g}\right)/2l_{fl}\right]=0 \end{array}$$

The second flying phase are as follows:

(10) $$\{\begin{array}{c} \begin{array}{c} \ddot{x}=0\\ \ddot{z}=-g \end{array}\\ \ddot{\beta }=0 \end{array}$$

The hind leg touch-down phase are as follows:

(1) Leading leg touches down are as follows:

(11) $$\{\begin{array}{c} m\ddot{x}-\left(F_{khl}+F_{chl}+F_{thrusthl}\right)\cos\left(\theta_{h}-\beta \right)+\tau_{hl}\sin\left(\theta_{h}-\beta \right)/l_{hl}=0\\ m\ddot{y}+\left(F_{khl}+F_{chl}+F_{thrusthl}\right)\sin\left(\theta_{h}-\beta \right)+\tau_{hl}\cos\left(\theta_{h}-\beta \right)/l_{hl}-mg=0\\ J\ddot{\beta }+\left(F_{khl}+F_{chl}+F_{thrusthl}\right)L\sin\theta_{h}/2-\left(1-L\cos\theta_{h}/2l_{hl}\right)\tau_{hl}=0 \end{array}$$

(2) Both legs touch down are as follows:

(12) $$\{\begin{array}{l} m\ddot{x}-\left(F_{khl}+F_{chl}+F_{thrusthl}\right)\cos\left(\theta_{h}-\beta \right)-\left(F_{khr}+F_{chr}+F_{thrusthr}\right)\cos\left(\theta_{h}-\theta_{g}-\beta \right)\\ +\tau_{hl}\sin\left(\theta_{h}-\beta \right)/l_{hl}+\tau_{hr}\sin\left(\theta_{h}-\theta_{g}-\beta \right)/l_{hr}=0\\ m\ddot{y}+\left(F_{khl}+F_{chl}+F_{thrusthl}\right)\sin\left(\theta_{h}-\beta \right)+\left(F_{khr}+F_{chr}+F_{thrusthr}\right)\sin\left(\theta_{h}-\theta_{g}-\beta \right)\\ +\tau_{hl}\cos\left(\theta_{h}-\beta \right)/l_{hl}+\tau_{hr}\cos\left(\theta_{h}-\theta_{g}-\beta \right)/l_{hr}-mg=0\\ J\ddot{\beta }+\left(F_{khl}+F_{chl}+F_{thrusthl}\right)L\sin\theta_{h}/2+\left(F_{khr}+F_{chr}+F_{thrusthr}\right)L\sin\left(\theta_{h}-\theta_{g}\right)/2\\ -\tau_{hl}\left(1-Lcos\theta_{h}/2l_{hl}\right)-\tau_{hr}\left[1-Lcos\left(\theta_{h}-\theta_{g}\right)/2l_{hr}\right]=0 \end{array}$$

(3) Following leg touches down are as follows:

(13) $$\{\begin{array}{c} m\ddot{x}-\left(F_{kfl}+F_{cfl}+F_{thrustfl}\right)\cos\left(\theta_{f}+\theta_{g}+\beta \right)+\tau_{fl}\sin\left(\theta_{f}+\theta_{g}+\beta \right)/l_{fl}=0\\ m\ddot{y}+\left(F_{kfl}+F_{cfl}+F_{thrustfl}\right)\sin\left(\theta_{f}+\theta_{g}+\beta \right)+\tau_{fl}\cos\left(\theta_{f}+\theta_{g}+\beta \right)/l_{fl}-mg=0\\ J\ddot{\beta }-\left(F_{kfl}+F_{cfl}+F_{thrustfl}\right)L\sin\left(\theta_{f}+\theta_{g}\right)/2-\tau_{fl}\left[1-Lcos\left(\theta_{f}+\theta_{g}\right)/2l_{fl}\right]=0 \end{array}$$

In the equations, $F_{chl}=k{\dot{L}}_{hlr}$ ; $F_{cfl}=k{\dot{L}}_{flr}$ ; $F_{chr}=k{\dot{L}}_{hrr}$ ; $F_{cfr}=k{\dot{L}}_{frr}$ ; $F_{khl}=k\left(L_{0}-L_{hlr}\right)$ ; $F_{kfl}=k\left(L_{0}-L_{flr}\right)$ ; $F_{khr}=k\left(L_{0}-L_{hrr}\right)$ ; $F_{kfr}=k\left(L_{0}-L_{frr}\right)$ ; $F_{thrusthl}$ is the active force of the hind leg (N); $F_{khl}$ is the spring force of the hind leg (N); $F_{chl}$ is the damping force of the hind leg (N); $\tau_{hl}$ is the active torque of the hip joint of the hind leg (N∙m); $F_{thrusthr}$ is the active force of the right hind leg (N); $F_{khr}$ is the spring force of the right hind leg (N); is the damping force of the right hind leg (N); $\tau_{hr}$ is the active torque of the hip joint of the right hind leg (N·m); $F_{thrustfl}$ is the active force of the left front leg (N); $F_{kfl}$ is the spring force of the left front leg (N); $F_{cfl}$ is the damping force of the left front leg (N); $\tau_{fl}$ is the active torque of the hip joint of the left front leg (N·m); $F_{thrustfr}$ is the active force of the right front leg (N); $F_{kfr}$ is the spring force of the right front leg (N); $F_{cfr}$ is the damping force of the right front leg (N); $\tau_{fr}$ is the active torque of the hip joint of the right front leg (N·m); $L_{hlr}$ is the actual length of the left hind leg (m); $L_{flr}$ is the actual length of the right front leg (m); $L_{hrr}$ is the actual length of the right hind leg (m); $L_{frr}$ is the actual length of the right front leg (m); $\theta_{g}$ is the angle between the leading leg and the following leg (rad).

## 3. High-Speed Running Gait Control Strategy Design

### 3.1. Double Flying Phase Bound Gait Control Strategy Design

#### 3.1.1. Control Strategy Based on the SLIP Model

The velocity control formula based on the SLIP model is as follows:

(14) $$l_{touch}=\frac{v_{x}T_{s}}{2}+k_{P}(v_{d}-v_{x})+k_{I}\sum_{step}\left(v_{d}-v_{x}\right)$$

In the equation, $k_{P}$ is the proportional term coefficient of the PI controller; $k_{I}$ is the integral term coefficient of the PI controller; $v_{d}$ is the expected speed (m/s); $v_{x}$ is the horizontal speed (m/s); $T_{s}$ is the touchdown cycle (s).

(15) $$\theta_{touch}=\arcsin\left(\frac{l_{touch}}{l_{0}}\right)+\beta$$

In the equation, $l_{0}$ is the expected length of the leg (m); β is the pitch angle (rad).

The height control based on the SLIP model can be viewed as a method of system total energy regulation:

(16) $$F_{thrust}=k_{P}\left(h_{d}-h_{max}\right)+k_{I}\sum_{step}\left(h_{d}-h_{max}\right)+F_{cc}$$

In the equation, $k_{P}$ is the proportional term coefficient of the PI controller; $k_{I}$ is the integral term coefficient of the PI controller; $h_{d}$ is the expected maximum height (m); $h_{\max}$ is the actual maximum height (m); $F_{cc}$ is a constant compensation term (N).

The pitch angle control based on the SLIP model can be formulated as follows:

(17) $$\tau =-k_{p}(\beta -\beta_{d})-k_{d}\dot{\beta }$$

In the equation, $k_{P}$ is the proportional term coefficient of the PD controller; $k_{d}$ is the coefficient of the differential term of the PD controller; $\beta_{d}$ is the expected pitch angle (rad); β is the actual pitch angle (rad).

#### 3.1.2. Double Flying Phase Bound Gait Control Strategy Based on the SLIP Model

**Figure 4:**
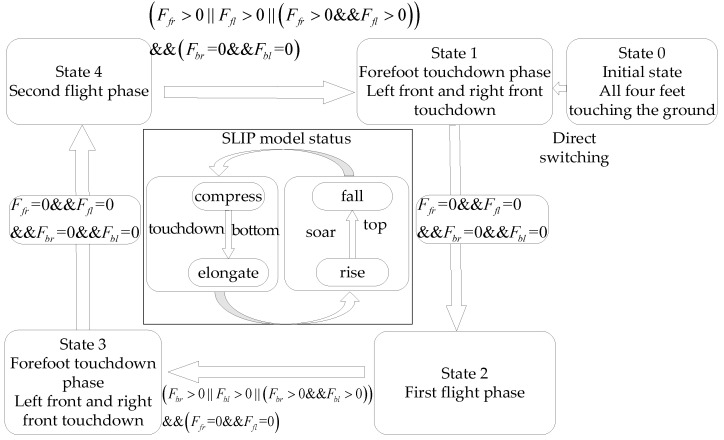
The various motion states and switching conditions of the double flying phase Bound gait.

Unlike the SLIP model, the three-bar model includes a longer body and pitch motion. Without controlling the body’s pitch angle, it may lead to divergence in the pitch angle over several running cycles, causing the robot to fall. Therefore, unlike the motion control of the SLIP model, effective control of the body’s pitch angle within the plane of the three-bar model during the double flying phase Bound gait cycle is necessary. The design of the double flying phase Bound gait state machine for the planar three-bar model uses a combined top-down and bottom-up approach, as shown in Figure 4.

Design the switching conditions between states to transition to the next motion state when the conditions are satisfied. During the double flying phase Bound gait running process, the sequence of leg movements for each state follows a loop from state 0 to states 1 through 4. Among these states, states 1 and 3 correspond to the foot-touching phases, while states 2 and 4 correspond to the aerial phases. According to the SLIP model’s motion states, the dynamic characteristics can be divided into three motion states, with states 1 and 2 corresponding to the foot-touching phases and state 3 corresponding to the aerial phase.

The formula for horizontal velocity control in the double flying phase Bound gait is as follows:

(18) $$\theta_{touch}=\arcsin\left(\frac{\frac{v_{x}T_{s}}{2}+k_{P}(v_{d}-v_{x})+k_{I}\sum_{step}\left(v_{d}-v_{x}\right)}{l_{0}}\right)+\beta$$

In the equation, $T_{s}$ is the time of first touchdown, and $T_{s}=\{\begin{array}{c} T_{n}=2\pi \sqrt{\frac{m}{k}}\begin{array}{cc} & n=1 \end{array}\\ T_{n}=T_{n-1}\begin{array}{cc} & n=2,3,4\ldots \end{array} \end{array}$ ; m is the weight of the center of mass (kg); k is the stiffness of the inverted leg (N/m); $\theta_{touch}$ is the angle of leg swing (rad); $v_{x}$ is the horizontal velocity (m/s); $k_{P}$ is the proportional term coefficient of the PI controller; $k_{I}$ is the integral term coefficient of the PI controller; $v_{d}$ is the expected speed (m/s); $\theta_{touch}$ is the angle of swinging the legs; $l_{0}$ is the expected length of the leg (m); β is the pitch angle (rad).

To achieve a more stable Bound gait running process, a method is employed to compensate for the leg’s thrust force and correct the robot’s pitch angle. In one gait cycle of the Bound gait, the maximum pitch angle when the front-side leg makes contact and compresses to the lowest point is defined as $\beta_{f}$ , and the maximum pitch angle when the hind-side leg makes contact and compresses to the lowest point is defined as $\beta_{y}$ . Using PID control to adjust the pitch angle leads to the following control formula:

(19) $$F_{p}=k_{p}\Delta \beta +k_{i}\sum_{step}\Delta \beta +k_{d}\Delta \dot{\beta }$$

In the equation, $k_{P}$ is the proportional coefficient of the PID controller; $k_{i}$ is the integral term coefficient of the PID controller; $k_{d}$ is the differential term coefficient of the PID controller.

The body height control formula is as follows:

(20) $$F_{thrust}=k_{P}\left(h_{d}-h_{max}\right)+k_{I}\sum_{step}\left(h_{d}-h_{max}\right)+F_{cc}$$

In the equation, $k_{P}$ is the proportional coefficient of the PI controller; $k_{I}$ is the integral term coefficient of the PI controller; $h_{d}$ is the expected maximum height (m); $h_{max}$ is the expected maximum height (m); $F_{cc}$ is the constant compensation term (N).

The thrust forces of the front-side leg and the hind-side leg are given by:

(21) $$\left\{ \begin{array}{l} F_{f}=k_{leg}\left(l_{0}-l_{freal}\right)-c_{leg}{\dot{l}}_{freal}+F_{thrust}+F_{p}\\ F_{h}=k_{leg}\left(l_{0}-l_{hreal}\right)-c_{leg}{\dot{l}}_{hreal}+F_{thrust}-F_{p} \end{array}\right.$$

In the equations, $k_{leg}$ is the leg stiffness (m/s); $c_{leg}$ is the leg damping (N·m/s); $l_{0}$ is the expected leg length (m); $l_{freal}$ is the actual leg length of the front side (m); $l_{hreal}$ is the actual leg length of the rear side (m).

Based on the designed state machine, the leg action sequence of the double flying phase Bound gait is determined, and the previous touchdown time is used to estimate the current touchdown time, with time is as follows:

(22) $$T_{sw}=T_{s}$$

In the equation, $T_{s}$ is the duration of last touchdown phase (s).

Using cubic spline to design the swing leg curve, the desired swing leg angle $a_{d}$ is as follows:

(23) $$a_{d}=a_{keep}+\left(\theta_{touch}-\beta -a_{keep}\right)\left[3{\left(T_{tr}/T_{sw}\right)}^{2}-2{\left(T_{tr}/T_{sw}\right)}^{3}\right]$$

In the equation, $a_{keep}$ is the angle between the legs and the body immediately after liftoff (rad); $\theta_{touch}$ is the expected touchdown angle (rad); β is the elevation angle of the fuselage (rad); $T_{tr}$ is the actual touchdown time (rad); $T_{sw}$ is the oscillation time (s).

The estimated aerial time is as follows:

(24) $$T_{\mathrm{ss}}=T_{s}+2T_{f}$$

In the equation, $T_{s}$ is the duration of the last touchdown phase(s); $T_{f}$ is the duration of the previous flight phase (s).

Design the desired length of the leg as follows:

(25) $$\{\begin{array}{c} l_{d}=l_{0}-\Delta l[3{\left(T_{sr}/T_{f}\right)}^{2}-2{\left(T_{sr}/T_{f}\right)}^{3}]\\ l_{d}=l_{0}-\Delta l\\ l_{d}=l_{0}+\Delta l\{3{\left[(T_{sr}-T_{s}-T_{f})/T_{f}\right]}^{2}-2{\left[(T_{sr}-T_{s}-T_{f})/T_{f}\right]}^{3}-1\} \end{array}\begin{array}{c} 0\leq T_{sr}<T_{f}\\ T_{f}\leq T_{sr}\leq T_{s}+T_{f}\\ T_{s}+T_{f}<T_{sr}\leq T_{ss} \end{array}$$

In the equations, $l_{0}$ is the original length of the leg (m); $T_{s}$ is the duration of the previous touchdown phase (s); $T_{f}$ is the duration of the previous flight phase (s); Δl is the shrinkage length (m); $T_{sr}$ is the actual lift time of the legs; $T_{ss}$ is the takeoff time (s).

### 3.2. Optimization of Rotatory Gallop Gait Model and Control Strategy Design

#### 3.2.1. D-SLIP Model Control Strategy

**Figure 5:**
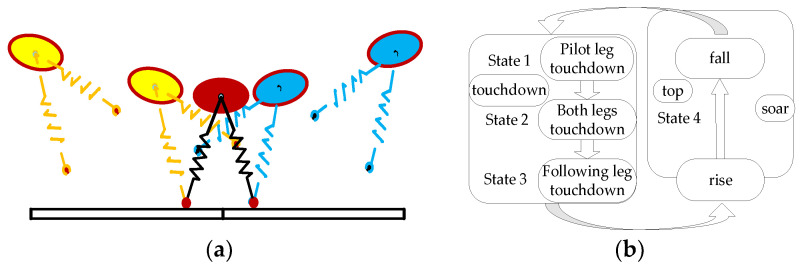
D-SLIP model. (**a**) The motion process of the D-SLIP model. (**b**) The motion states of the D-SLIP model.

The traditional SLIP model is a single-leg model and cannot be directly applied to the asymmetric Rotatory gallop gait. Therefore, it is necessary to optimize the traditional SLIP model with reference to the leg movement characteristics of the Gallop gait. The improved SLIP model has two supporting spring-damper legs, and it is defined as the D-SLIP model. Similar to the traditional SLIP model, the motion process of the D-SLIP model is divided into two phases: the aerial phase and the touch-down phase. However, during the touch-down process, the D-SLIP model is divided into three stages similar to the single-side leg touch-down process of the Gallop gait: leading leg touch-down; both legs touch-down; and following leg touch-down. The motion process of the D-SLIP model is shown in Figure 5a, and the state machine is shown in Figure 5b. The D-SLIP model can be directly used by the robot as an essential model for the legs on the same side. Through top-level control strategy coordination, the robot’s Gallop gait can be completed.

The dynamic analysis of the D-SLIP model is completed, and the dynamic equations for the aerial phase are as follows:

(26) $$\{\begin{array}{c} \ddot{x}=0\\ \ddot{z}=-g\\ \ddot{\beta }=0 \end{array}$$

Equations for the touch-down phase are as follows:

(1) Leading leg touch-down stage are as follows:

(27) $$\{\begin{array}{c} m\ddot{x}-k(L_{0}-L_{l})\frac{\sqrt{L_{l2}-Z_{r2}}}{L_{l}}=0\\ m\ddot{z}-k(L_{0}-L_{l})\frac{Z_{r}}{L_{l}}+mg=0\\ J\ddot{\beta }+\tau_{l}=0 \end{array}$$

(2) Both legs touch-down stage are as follows:

(28) $$\{\begin{array}{c} m\ddot{x}-k(L_{0}-L_{l})\frac{\sqrt{L_{l2}-Z_{r2}}}{L_{l}}-k(L_{0}-L_{f})\frac{\sqrt{L_{f2}-Z_{r2}}}{L_{f}}=0\\ m\ddot{z}-k(L_{0}-L_{l})\frac{Z_{r}}{L_{l}}-k(L_{0}-L_{f})\frac{Z_{r}}{L_{f}}+mg=0\\ J\ddot{\beta }+\tau_{l}+\tau_{f}=0 \end{array}$$

(3) Following leg touch-down stage are as follows:

(29) $$\{\begin{array}{c} m\ddot{x}-k(L_{0}-L_{f})\frac{\sqrt{L_{f2}-Z_{r2}}}{L_{f}}=0\\ m\ddot{z}-k(L_{0}-L_{f})\frac{Z_{r}}{L_{f}}+mg=0\\ J\ddot{\beta }+\tau_{l}=0 \end{array}$$

In the equations, x is the horizontal displacement (m); z is the vertical displacement (m); m is the centroid mass (kg); g is the gravity acceleration (m/s²); $L_{l}$ is the actual length of the leading leg (m); $L_{f}$ is the actual length of the following leg (m); $L_{0}$ is the original length of the leg (m); $Z_{r}$ is the actual height of the center of mass (m); k is the leg stiffness (N/m).

The force on the body at touchdown is shown in Equation (30).

(30) $$\{\begin{array}{c} \sum F_{x}=F_{l}\sin\theta_{gl}-F_{f}\sin\theta_{gf}\\ \sum F_{z}=F_{l}\cos\theta_{gl}+F_{f}\cos\theta_{gf}-mg \end{array}$$

In the equations, $\Delta l_{l}=l_{0}-\frac{Z_{r}}{\cos\theta_{gl}}$ ; $\Delta l_{f}=l_{0}-\frac{Z_{r}}{\cos\theta_{gf}}$ ; $F_{l}=\left\{ \begin{array}{l} k\Delta l_{l}\\ 0 \end{array}\right.\begin{array}{c} \Delta l_{l}>0\\ \Delta l_{l}=0 \end{array}$ ; $F_{f}=\left\{ \begin{array}{l} k\Delta l_{f}\\ 0 \end{array}\right.\begin{array}{c} \Delta l_{f}>0\\ \Delta l_{f}=0 \end{array}$ ; $\theta_{g}=\theta_{gl}+\theta_{gf}$ ; $\theta_{gl}$ is the angle between the leading leg and the virtual leg (rad); $\theta_{gf}$ is the angle between the following leg and the virtual leg (rad); m is the centroid mass (kg); $\Delta l_{f}$ is the change in length of the following leg (m); $\Delta l_{l}$ is the change in length of the leading leg (m); $Z_{r}$ is the real-time height of the pivot point (m). k is the leg stiffness (N/m).

The horizontal distance between the neutral point and the pivot point is $l_{zero}$ :

(31) $$l_{zero}=\frac{v_{x}T_{s}}{2}$$

In the equation, $v_{x}$ is the actual speed (m/s); $T_{s}$ is the touchdown time (s).

To ensure accurate touchdown angles, real-time measurement of the body height $Z_{rt}$ is necessary. By combining real-time height and the horizontal distance to the neutral point with a velocity closed-loop PI controller, an equivalent leg swing angle $\theta_{touch}$ can be obtained as follows:

(32) $$\theta_{touch}=\arctan\left(\frac{l_{zero}}{Z_{r}}\right)+k_{P}(v_{d}-v_{x})+k_{I}\sum_{step}\left(v_{d}-v_{x}\right)+\beta$$

In the equation, $v_{x}$ is the actual speed (m/s); $T_{s}$ is the touchdown time (s); $k_{P}$ is the proportional term coefficient of the PI controller; $k_{I}$ is the integral term coefficient of the PI controller; $v_{d}$ is the expected speed (m/s); $Z_{r}$ is the actual height of the hinge point (m); β is the pitch angle (rad).

Obtain the touch-down angle of the leading leg $\theta_{lsw}$ and the touch-down angle of the following leg $\theta_{fsw}$ , as follows:

(33) $$\left\{ \begin{array}{l} \theta_{lsw}=\theta_{touch}-\frac{\theta_{g}}{2}\\ \theta_{fsw}=\theta_{touch}+\frac{\theta_{g}}{2} \end{array}\right.$$

In the equation, $\theta_{g}$ is the angle between the leading leg and the following leg (rad); $\theta_{touch}$ is the expected swing angle of the equivalent leg (rad).

The height control of the D-SLIP model is the same as the traditional SLIP model control strategy, with the formula as follows:

(34) $$F_{thrust}=k_{P}\left(h_{d}-h_{max}\right)+k_{I}\sum_{step}\left(h_{d}-h_{max}\right)+F_{cc}$$

In the pitch control of the D-SLIP model, the body angle servo control occurs during the both-legs touch-down stage in the touch-down phase, and the applied torque is as follows:

(35) $$\tau =-k_{p}(\beta -\beta_{d})-k_{d}\dot{\beta }$$

In the equation, $k_{P}$ is the proportional term coefficient of the PI controller; $k_{I}$ is the integral term coefficient of the PI controller; $h_{d}$ is the expected maximum height (m); $h_{\max}$ is the expected maximum height (m); $F_{cc}$ is the constant compensation term (N).

#### 3.2.2. Control of Rotatory Gallop Gait Based on the D-SLIP Model

**Figure 6:**
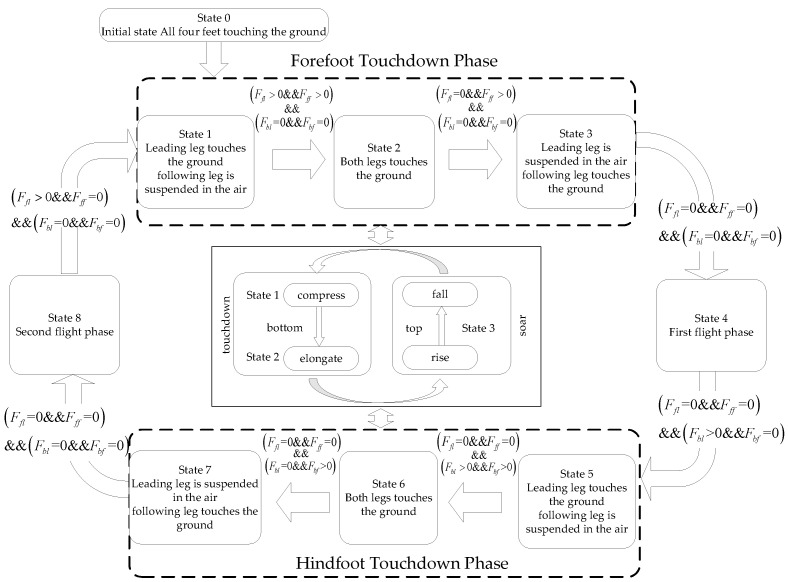
The various motion states and switching conditions of the Rotatory gallop gait.

In the Rotatory gallop gait, the quadrupeds make contact with the ground in a specific sequence. The front and hind legs can be treated as two groups of D-SLIP models. During the running process, the top-level control algorithm coordinates the control of the two sets of models to achieve the Rotatory gallop gait in the bio-inspired robot prototype. A state machine can be designed as shown in Figure 6.

To estimate the touch-down angle, the real-time height of the front and hind hinge points needs to be calculated as follows:

(36) $$h_{rtj}=\{\begin{array}{c} h_{rt}+L\sin\beta /2\\ h_{rt}-L\sin\beta /2 \end{array}\begin{array}{c} state=4\\ state=8 \end{array}$$

In the equations, L is the length of the fuselage (m); $h_{rt}$ is the height of the fuselage (m); state is the state of motion.

Define the equivalent leg swing angle $\theta_{touch}$ as follows:

(37) $$\theta_{touch}=\arctan\left(\frac{v_{x}T_{st}}{2h_{rtj}}\right)+k_{P}(v_{d}-v_{x})+k_{I}\sum_{step}\left(v_{d}-v_{x}\right)+\beta$$

In the equation, $k_{P}$ is the proportional term coefficient of the PI controller; $k_{i}$ is the integral term coefficient of the PD controller; $v_{d}$ is the expected speed (m/s); $Z_{r}$ is the actual height of the hinge point (m); β is the pitch angle (rad).

The leading leg swing angle $\theta_{lsw}$ and the following leg swing angle $\theta_{fsw}$ are defined as follows:

(38) $$\left\{ \begin{array}{l} \theta_{lsw}=\theta_{touch}-\frac{\theta_{g}}{2}\\ \theta_{fsw}=\theta_{touch}+\frac{\theta_{g}}{2} \end{array}\right.$$

In the equations, $\theta_{g}$ is the angle between the leading and the following legs (rad).

The pitch angle can be controlled using the following formula:

(39) $$F_{p}=k_{p}\Delta \beta +k_{i}\sum_{step}\Delta \beta +k_{d}\Delta \dot{\beta }$$

In the equations, $k_{P}$ is the proportional term coefficient of the PID controller; $k_{i}$ is the integral term coefficient of the PID controller; $k_{d}$ is the differential coefficient of the PID controller; β is the pitch angle (rad).

The body height control formula is:

(40) $$F_{thrust}=k_{P}\left(h_{d}-h_{max}\right)+k_{I}\sum_{step}\left(h_{d}-h_{max}\right)+F_{cc}$$

In the equation, $k_{P}$ is the proportional term coefficient of the PI controller; $k_{i}$ is the integral term coefficient of the PI controller; $h_{d}$ is the expected maximum height (m); $h_{\max}$ is the actual maximum height (m); $F_{cc}$ is the constant compensation term (N).

In summary, the thrust of the left front leg $F_{fl}$ , the right front leg $F_{fr}$ , the left rear leg $F_{hl}$ , and the right rear leg $F_{hr}$ can be expressed as follows:

(41) $$\left\{ \begin{array}{l} F_{fl}=k_{leg}\left(l_{0}-l_{flreal}\right)-c_{leg}{\dot{l}}_{flreal}+\frac{1}{2}F_{thrust}+\frac{1}{2}F_{p}\\ F_{fr}=k_{leg}\left(l_{0}-l_{frreal}\right)-c_{leg}{\dot{l}}_{frreal}+\frac{1}{2}F_{thrust}+\frac{1}{2}F_{p}\\ F_{hl}=k_{leg}\left(l_{0}-l_{hlreal}\right)-c_{leg}{\dot{l}}_{hlreal}+\frac{1}{2}F_{thrust}-\frac{1}{2}F_{p}\\ F_{hr}=k_{leg}\left(l_{0}-l_{hrreal}\right)-c_{leg}{\dot{l}}_{hrreal}+\frac{1}{2}F_{thrust}-\frac{1}{2}F_{p} \end{array}\right.$$

In the equations, $k_{i}$ is the leg stiffness (m/s); $c_{leg}$ is the leg damping (N·s/m); $l_{0}$ is the desired leg length (m); $l_{flreal}$ is the actual length of the left front leg (m); $l_{frreal}$ is the actual length of the right front leg (m); $l_{hlreal}$ is the actual length of the left rear leg (m); $l_{hrreal}$ is the actual length of the right rear leg (m).

The swing strategy for the Rotatory gallop gait is similar to the double flying phase Bound gait, with a swing time $T_{sw}$ defined as follows:

(42) $$T_{sw}=T_{s}$$

The desired swing angles of the leading leg $a_{ld}$ and the following leg $a_{fd}$ are defined as follows:

(43) $$a_{d}=a_{keep}+\left(\theta_{touch}-\beta -a_{keep}\right)\left[3{\left(T_{tr}/T_{sw}\right)}^{2}-2{\left(T_{tr}/T_{sw}\right)}^{3}\right]$$

(44) $$\left\{ \begin{array}{l} a_{ld}=a_{d}-\frac{1}{2}\theta_{g}\\ a_{fd}=a_{d}+\frac{1}{2}\theta_{g} \end{array}\right.$$

In the equations, $a_{keep}$ is the equivalent leg–body angle at liftoff (rad); $\theta_{touch}$ is the desired touch-down angle of the equivalent leg (rad); β is the elevation angle of the fuselage (rad); $T_{tr}$ is the actual touchdown time (s); $\theta_{g}$ is the angle between the leading leg and the following leg (rad).

The aerial time between the end of one ground contact phase and the next ground contact phase can be estimated as follows:

(45) $$T_{\mathrm{ss}}=T_{s}+2T_{f}$$

In the equation, $T_{s}$ is the duration of last touchdown phase (s); $T_{f}$ is the duration of the previous flight phase (s).

The desired leg length is designed as follows:

(46) $$\{\begin{array}{c} l_{d}=l_{0}-\Delta l[3{\left(T_{sr}/T_{f}\right)}^{2}-2{\left(T_{sr}/T_{f}\right)}^{3}]\\ l_{d}=l_{0}-\Delta l\\ l_{d}=l_{0}+\Delta l\{3{\left[(T_{sr}-T_{s}-T_{f})/T_{f}\right]}^{2}-2{\left[(T_{sr}-T_{s}-T_{f})/T_{f}\right]}^{3}-1\} \end{array}\begin{array}{c} 0\leq T_{sr}<T_{f}\\ T_{f}\leq T_{sr}\leq T_{s}+T_{f}\\ T_{s}+T_{f}<T_{sr}\leq T_{ss} \end{array}$$

In the equations, $l_{0}$ is the original length of the leg (m); Δl is the shrinkage length (m); $T_{sr}$ is the actual leg lift time (s).

## 4. Simulation Verification

To validate the correctness of the control algorithm, the Adams2020 is used to construct the dynamic prototype, and the MATLAB 2020b/Simulink is used to create a virtual prototype control system. The two software platforms are connected for combined simulation. The contact between the virtual prototype and the ground is selected from the collision model in the Adams model, and the communication period between MATLAB/Simulink and Adams is 1 ms. Adams outputs the horizontal displacement, vertical displacement, pitch angle, foot–ground contact force of the virtual prototype with respect to the ground, as well as the actual leg length and the actual pendulum angle of the model itself and the control system constructed in Simulink is controlled by the output signals from Adams to the virtual prototype through the MATLAB/Simulink. The control system built by Simulink controls the motion of the virtual prototype through the output signals of Adams.

### 4.1. Simulation of SLIP Model and D-SLIP Model

To verify the motion control methods of the SLIP model, a virtual prototype of the SLIP model is built, allowing the body to pitch and move within a 2D(Dimensionality) plane. The body and leg articulate at the hip joint, and the leg can swing forward and backward. The leg consists of upper and lower parts connected by a moving joint, allowing vertical movement. The model parameters are shown in Table 1.

**Figure 7:**
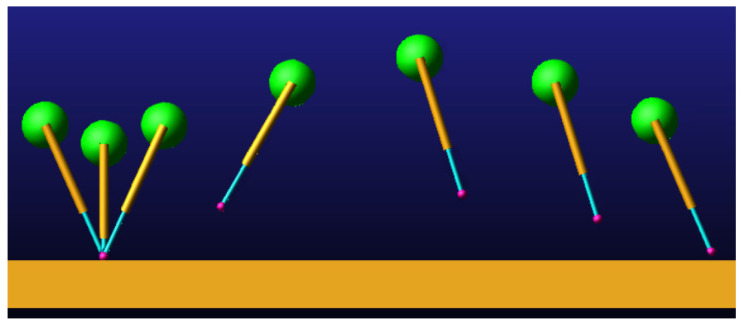
SLIP model simulation motion screenshot.

During the model simulation motion, the visualization of the prototype uniform speed motion process is shown in Figure 7. The model compresses and extends the leg during the ground contact phase and swings the leg during the aerial phase.

**Figure 8:**
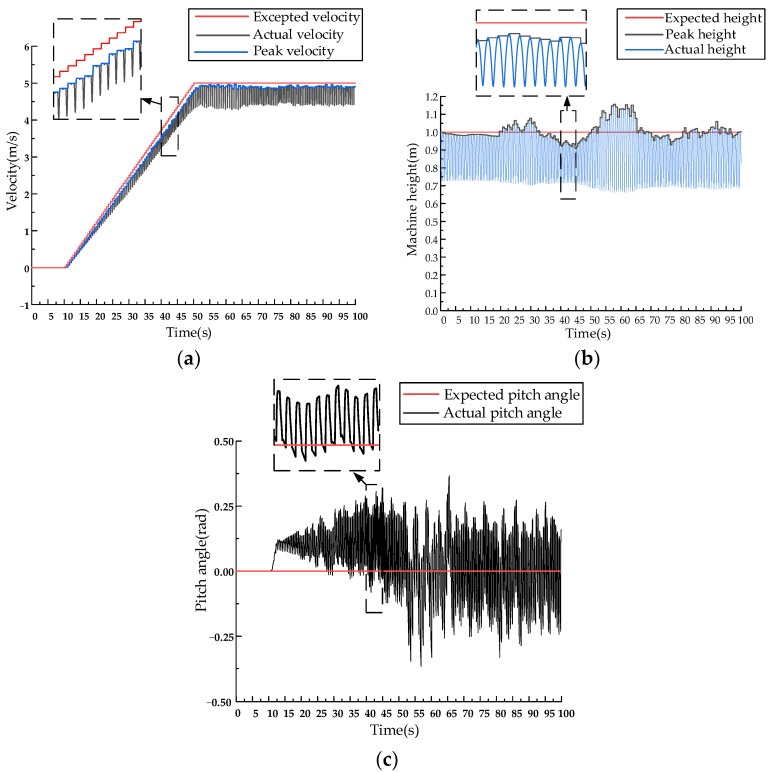
SLIP model simulation curve. (**a**) SLIP model speed curve. (**b**) SLIP model fuselage height curve. (**c**) SLIP model pitch angle curve.

The system starts from a static state. In the first 10 s of the simulation, the expected velocity of the inverted pendulum is zero, and it jumps in place. Speed, pitch, and body height fluctuated very little during the 10 s and stayed close to the desired values. After 10 s, the system starts to accelerate and reaches a speed of 5 m/s at 50 s, maintaining this speed for the next 50 s. The velocity control curve, body height control curve, and pitch angle control curve of the SLIP model are shown in Figure 8.

To validate the motion control methods of the D-SLIP model, a virtual prototype of the D-SLIP model is built. The body can pitch and move within a 2D plane. The left hip joint of the body is articulated to the left leg, and the right hip joint is articulated to the right leg. Each leg is divided into upper and lower parts connected by a moving joint, allowing vertical movement. Torque applied simultaneously to the left and right hip joints between the body and the left and right legs, and a force applied between the upper and lower legs is set between the upper and lower legs. The model parameters are shown in Table 2.

**Figure 9:**
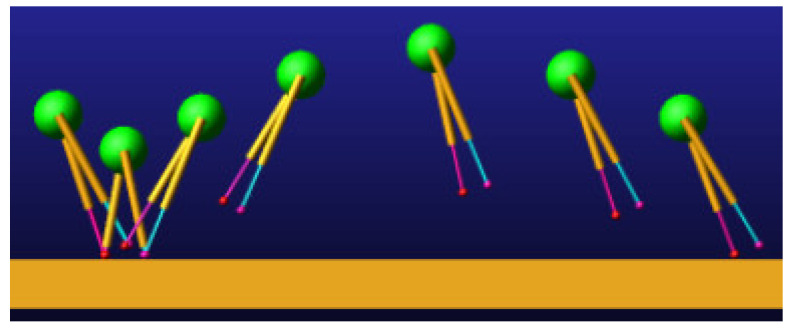
D-SLIP model simulation motion screenshot.

During the model simulation motion, the visualization of the prototype uniform speed motion process is shown in Figure 9.

**Figure 10:**
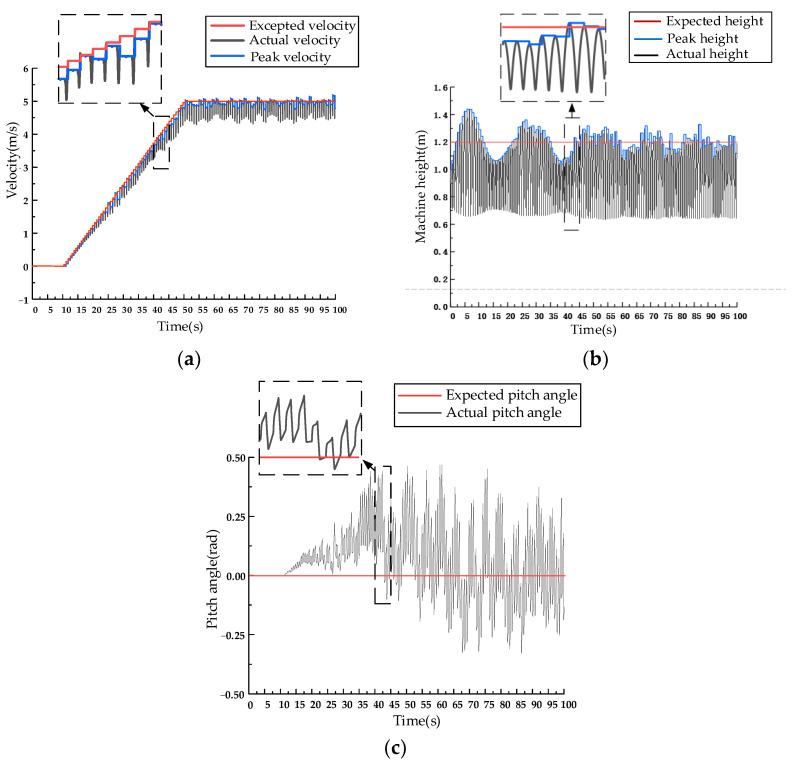
D-SLIP model simulation curve. (**a**) D-SLIP model speed curve. (**b**) D-SLIP model height curve. (**c**) D-SLIP model pitch angle curve.

The system starts from a static state, and for the first 10 s, the expected velocity of the D-SLIP model is zero, and the machine jumps in place. After 10 s, the system starts to accelerate and reaches a speed of 5 m/s at 50 s, maintaining this speed for the next 50 s. The velocity control curve, body height control curve, and pitch angle control curve of the D-SLIP model are shown in Figure 10.

The combined simulation system successfully achieves the continuous stable running of the virtual prototypes of the SLIP and D-SLIP models, confirming the feasibility of the designed control algorithms.

### 4.2. Simulation of Double Flying Phase Bound Gait

**Figure 11:**
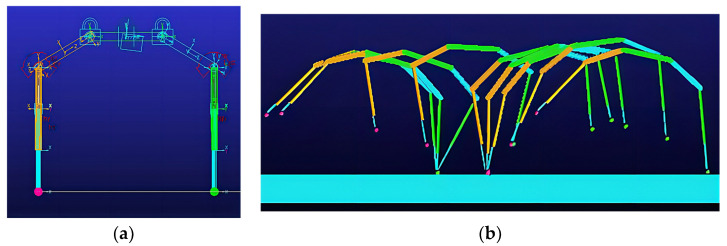
Double flying phase Bound gait. (**a**) Virtual prototype. (**b**) Screenshot of simulated motion.

A virtual prototype is constructed, as shown in Figure 11a. The body can pitch and move within a 2D plane. The body and legs articulate at the hip joints, and the legs can swing forward and backward. Each leg is divided into upper and lower parts connected by a moving joint, allowing vertical movement. The hip joint applies torque to both the body and the leg, and forces are applied between the upper and lower parts of each leg. The front and hind segments of the spine are hinged to the front and hind legs, respectively, and restrained by the rotating vice. The model parameters are shown in Table 3.

During the simulation process of the uniform motion, the visualization of the prototype with the Bound gait uniform speed motion process is shown in Figure 11b.

**Figure 12:**
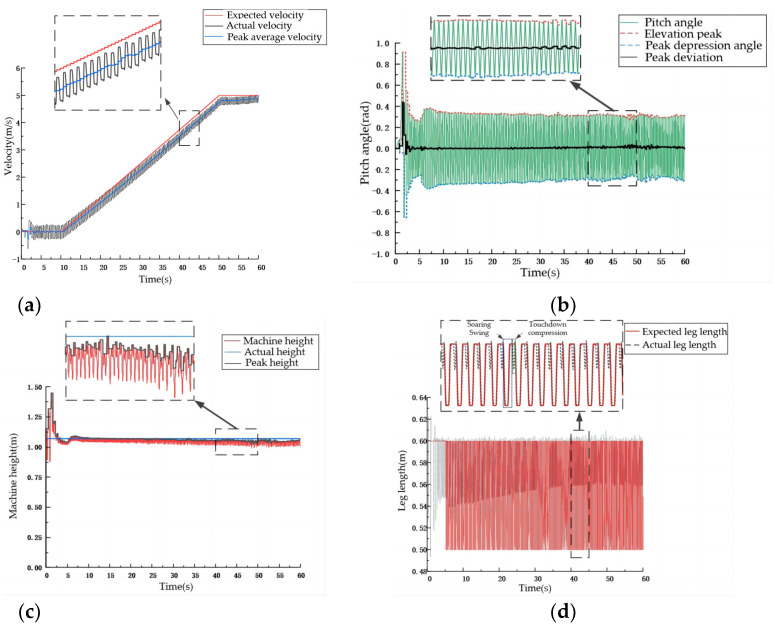
Double flying phase Bound gait simulation dynamic curve. (**a**) Speed control curve. (**b**) Pitch angle control curve. (**c**) Fuselage height control curve. (**d**) Leg length control curve.

The prototype completes the Bound gait takeoff within the first 10 s and controls the forward speed to 0 m/s for stationary jumping. From 10 s to 50 s of simulation time, the prototype accelerates from 0 m/s to 5 m/s for stable running. This results in velocity control curves, pitch control curves, body height control curves using compensation thrust, and leg length control curves, as shown in Figure 12.

The continuous stable running of the virtual prototype is achieved through a combined simulation system, confirming the feasibility of the Bound gait control algorithm based on the alternate SLIP model.

### 4.3. Rotatory Gallop Gait Simulation

**Figure 13:**
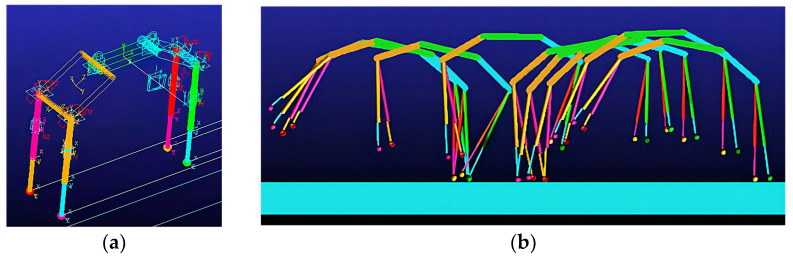
Rotatory gallop gait. (**a**) Virtual prototype. (**b**) Screenshot of simulated motion.

The Rotatory gallop gait virtual prototype is built as shown in Figure 13a. The virtual prototype can perform pitch motion and movement in a two-dimensional plane, and the prototype body is composed of three segments of the spine. In order to achieve coordinated control of the four legs, the front and hind segments of the spine are designed with different widths, and the three segments of the spine are fixed and connected to treat the entire fuselage as a rigid body. The front and hind segments of the spine connect four legs, each consisting of upper and lower parts constrained by rotary joints. The model parameters are shown in Table 4. 

During the simulation process of the uniform motion, the visualization of the prototype with the Rotatory gallop gait uniform speed motion process is shown in Figure 13b.

**Figure 14:**
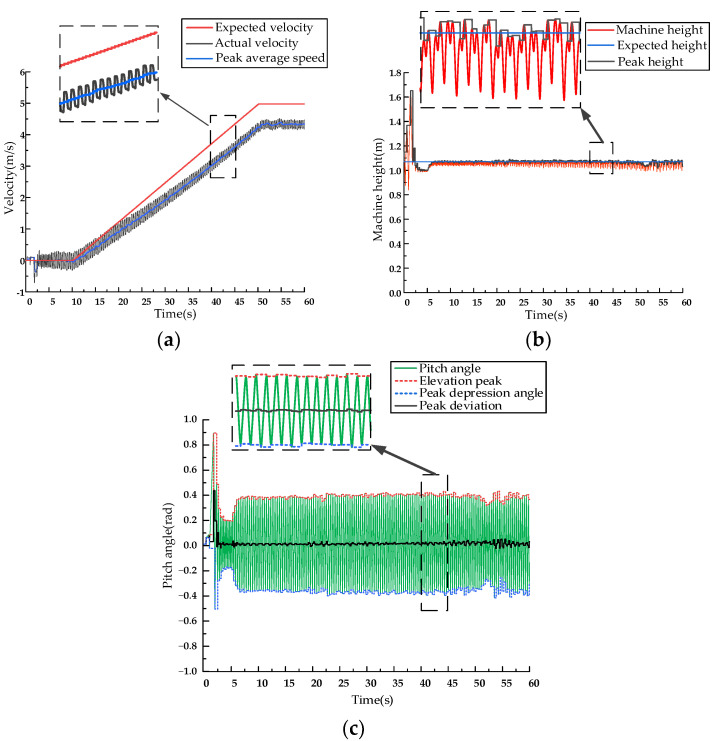
Rotatory gallop gait dynamic curve. (**a**) Prototype speed control curve. (**b**) Fuselage height control curve. (**c**) Pitch angle control curve.

The prototype completes the Gallop gait takeoff within the first 10 s and controls the forward speed to 0 m/s for stationary jumping. From 10 s to 50 s of simulation time, the prototype accelerates from 0 m/s to 5 m/s for stable galloping. The speed control curve, body height control curve, and pitch control curve of the prototype are shown in Figure 14.

The virtual prototype’s continuous stable Gallop gait running is achieved through a combined simulation system, confirming the feasibility of the Gallop gait control algorithm based on the alternate D-SLIP model.

## 5. Experimental Verification

### 5.1. Design of Robot Leg Control Method

**Figure 15:**
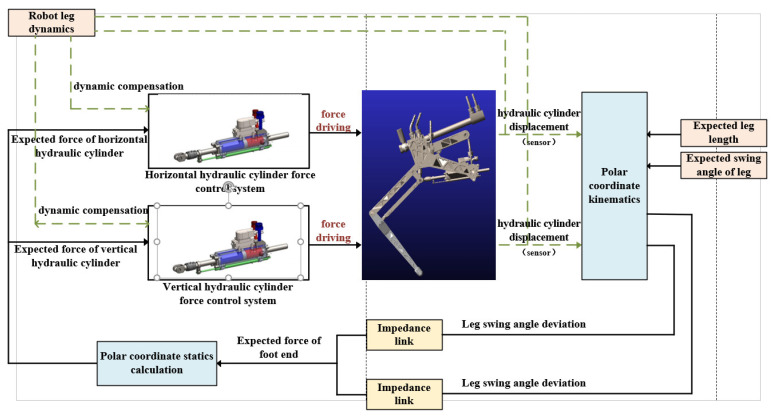
Robot single leg control diagram.

Mammals provide power to the legs through muscles and control the movement position and output force of the legs through the sensing system. Similarly, the robot uses a hydraulic cylinder to provide power for the leg and senses the motion position and output force through displacement and force sensors. Combined with the above two parts of the control strategy, based on the bionic principle, the leg motion control diagram was designed as shown in Figure 15 [29,30].

It can be seen from the leg motion diagram described in Figure 15 that the hydraulic cylinder force control system in the leg motion control is the impedance control inner loop, which applies the active force to drive the virtual prototype directly. The expected force of the hydraulic cylinder force control system is determined by the impedance control outer ring according to the deviation of the expected length of the leg, the swing angle, and the actual length and the swing angle. After the impedance link determines the expected force of the foot end, the polar coordinate statics solution can provide the expected force for the inner ring of the horizontal and vertical hydraulic drive unit force control system. The control system combines impedance control and hydraulic cylinder force control to realize the leg based on force impedance control. At the same time, in order to eliminate the interference caused by the dynamics of its own mechanical structure, this paper introduces the dynamic compensation of the leg system into the existing robot leg based on the force impedance control system to improve the accuracy of the robot leg based on the force impedance control system.

### 5.2. High-Speed Running Robot Prototype Introduction

**Figure 16:**
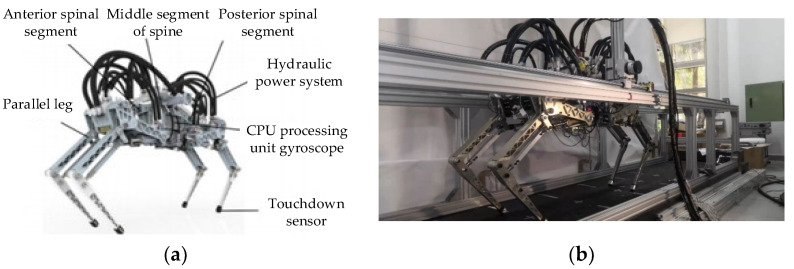
High-speed running robot prototype. (**a**) High-definition rendering. (**b**) Actual prototype.

As shown in Figure 16, the experimental prototype of the high-speed quadruped robot developed in this study is presented. The prototype mainly consisted of the body mechanical structure, leg mechanical structure, hydraulic power system, environmental perception sensors, and control system. The aluminum alloy profiles were used to create a running environment, enabling the robot to perform walking and running tests on a flat surface. The robot was constrained to a pair of horizontal rails by supports placed on both sides of the body and connected to the body’s axis. It was then connected to a treadmill placed below the constraint platform. The selected treadmill was an elongated type (5 m in length with an effective stroke of 4.5 m) designed for animal use, which meets the running requirements of the robot.

### 5.3. High-Speed Robot Running Experiment

**Figure 17:**
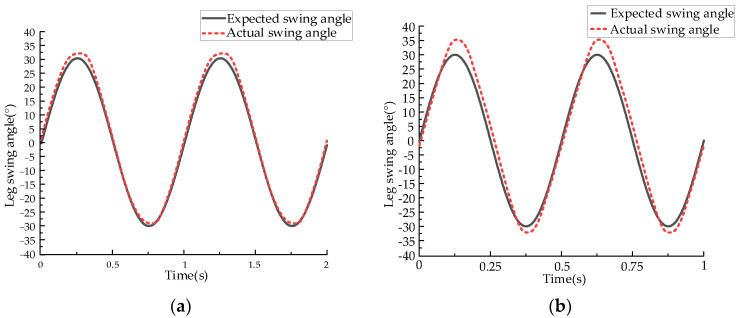
Swing leg control curve. (**a**) The experimental curve under 1 Hz working condition. (**b**) The experimental curve under 2 Hz working condition.

To achieve effective running control, performance testing experiments were conducted on the prototype. Accurate servo control of the leg swing angle is the foundation for controlling the forward speed through the touchdown angle. Therefore, experimental research was conducted on the servo control system of the robot’s leg swing angle. Leg-swinging experiments were performed at frequencies of 1 Hz and 2 Hz, and the experimental curves are shown in Figure 17.

The experimental results demonstrate that the robot’s leg exhibits good position servo control effects at frequencies of 1–2 Hz. Although there is a slight overshoot in the follow-up curve, the actual swing angle curve aligns closely with the target curve. This indicates that the robot’s leg meets the requirements for high-speed running.

**Figure 18:**
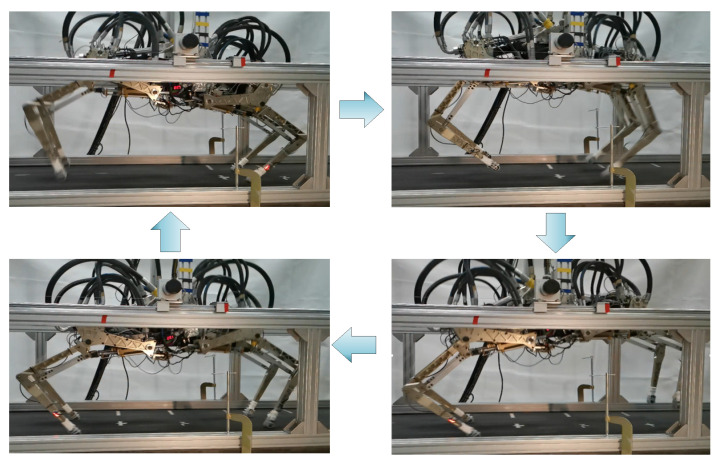
Screenshot of running experiment.

After completing the leg swing verification experiments, the running control of the Bound gait was conducted on the existing high-speed robot platform. Screenshots of the running process are shown in Figure 18.

**Figure 19:**
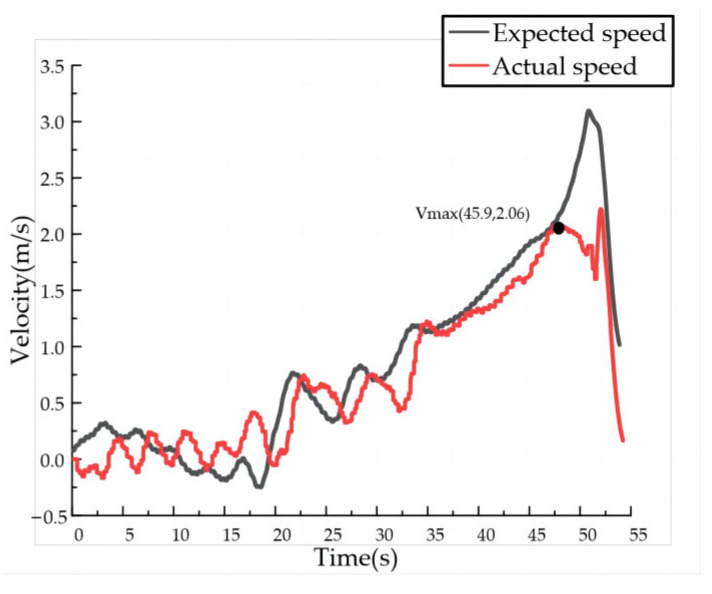
Running speed servo curve.

**Figure 20:**
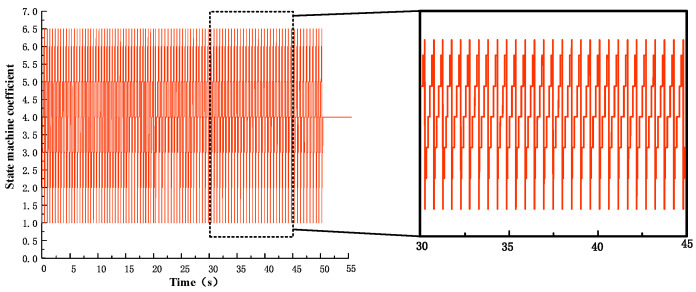
Output coefficient of the state machine.

**Figure 21:**
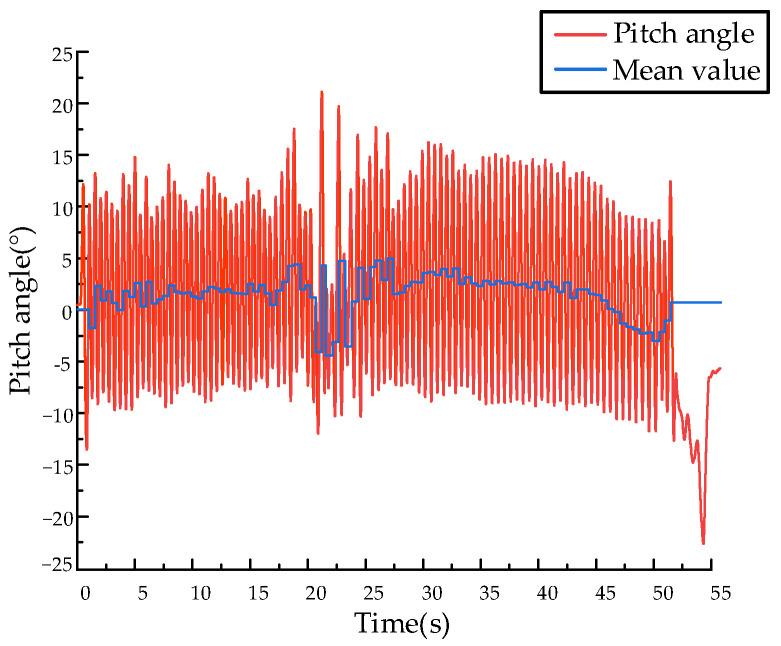
Pitch angle motion curve.

The velocity tracking curve is shown in Figure 19. The quadruped robot jumped in place after completing the Bound gait takeoff motion. After the output of the state machine stabilized, it continuously accelerated the entire machine by controlling the foot contact angle based on the deviation between the input expected speed and the actual speed. At 46 s, it accelerated to a horizontal running speed of 2 m/s. As shown in Figure 20, the output coefficient of state machine law during the running process. The motion states of the robot are well connected, and the state machine will not be disordered during the acceleration process. The pitch motion curve and the extreme and mean values of pitch angle during running are shown in Figure 21. The robot exhibited regular pitch motion during running, with minor fluctuations in the average pitch angle around 0° during acceleration, indicating the absence of instability caused by divergence of the pitch angle. In summary, the double flying phase Bound gait of the high-speed robot prototype is stable, and the control system possesses certain robustness.

## 6. Conclusions

In response to the high-speed running requirements of quadruped robots, this paper, based on the SLIP and optimized D-SLIP models, designed running control strategies for the common double flying phase Bound gait and Rotatory gallop gait. Through virtual prototype simulations, the correctness of the SLIP model, D-SLIP model, double flying phase Bound gait, and Rotatory gallop gait control methods were verified, achieving high-speed running for quadruped robots. Stable running of the double flying phase Bound gait was achieved on the existing high-speed robot platform, with a running speed of up to 2 m/s, and the control system possesses certain robustness.

## Figures and Tables

**Table 1:** The parameters of the SLIP model.

Parameter	Value	Unit
Mass of the body	50	kg
Rotational damper	0.3	N·m·s/rad
Upper leg mass	0.3	kg
Lower leg mass	0.2	kg
Translational damper	15	N·s/m
Foot contact force stiffness	1000	MN/m
Static friction coefficient	1	\
Dynamic friction coefficient	0.8	\
Velocity control PID parameters	1.1, 0, 0.2	\
Fuselage altitude control PID parameters	10, 60, 0	\
Pitch angle control PID parameters	50, 40, 0	\
Leg stiffness	15	N/mm
Leg original length	0.6	m
Height setpoint thrust	140	N

**Table 2:** The parameters of the D-SLIP model.

Parameter	Value	Unit
Mass of the body	50	kg
Damper for left and right hip joint rotation	0.15	N·m·s/rad
Mass of the upper part of the left and right leg	0.15	kg
Mass of the lower part of the left and right leg	0.1	kg
Damper for left and right leg movement	15	N·s/m
Foot contact force stiffness	1000	MN/m
Static friction coefficient	1	\
Dynamic friction coefficient	0.8	\
Lead, follow leg angle	π/18	rad
Velocity control PID parameters	1.5, 0.05, 0	\
Fuselage altitude control PID parameters	60, 120, 0	\
Pitch angle control PID parameters	70, 200, 5	\
Leg stiffness	15	N/mm
Leg original length	0.6	m
Height setpoint thrust	300	N

**Table 3:** The parameters of double flying phase Bound gait model.

Parameter	Value	Unit
Mass of the body	75	kg
Damper for front and hind hip joint rotation	0.6	N·m·s/rad
Mass of the upper part of the front and hind leg	0.6	kg
Mass of the lower part of the front and hind leg	0.4	kg
Damper for front and hind leg movement	30	N·s/m
Foot contact force stiffness	1000	MN/m
Static friction coefficient	1	\
Dynamic friction coefficient	0.8	\
Velocity control PID parameters	4, 0.3, 0	\
Fuselage altitude control PID parameters	0.35, 0.8, 0	\
Pitch angle control PID parameters	0.2, 1.5, 0	\
Leg stiffness	15	N/mm
Leg original length	0.6	m
Height setpoint thrust	500	N

**Table 4:** The parameters of the Rotatory gallop gait model.

Parameter	Value	Unit
Mass of the body	75	kg
Damper for front and hind hip joint rotation	0.3	N·m·s/rad
Upper leg mass	0.3	kg
Lower leg mass	0.2	kg
Translational damper	15	N·s/m
Foot contact force stiffness	1000	MN/m
Static friction coefficient	1	\
Dynamic friction coefficient	0.8	\
Lead, follow leg angle	Pi/18	rad
Velocity control PID parameters	2.5, 0.01, 0.2	\
Fuselage altitude control PID parameters	2, 4, 0	\
Pitch angle control PID parameters	0.5, 1.5, 0	\
Leg stiffness	10	N/mm
Leg original length	0.6	m
Height setpoint thrust	500	N

## Data Availability

Data are contained within the article.
